# Diagnostic value of procalcitonin for bacterial infections in patients undergoing hemodialysis: a systematic review and meta-analysis

**DOI:** 10.1080/0886022X.2021.2021236

**Published:** 2022-02-15

**Authors:** Mei Tao, Danna Zheng, Xudong Liang, Qiang He, Wei Zhang

**Affiliations:** a Department of Nephrology, The Affiliated Hospital of Hangzhou Normal University, Zhejiang, PR China; b Department of Nephrology, Zhejiang Provincial People’s Hospital and Affiliated People’s Hospital, Hangzhou Medical College, Zhejiang, PR China; c Chinese Medical Nephrology Key Laboratory of Zhejiang Province, Hangzhou, PR China

**Keywords:** Procalcitonin, renal dialysis, C-reactive protein, bacterial infections, sensitivity and specificity

## Abstract

**Background:**

The diagnostic value of procalcitonin (PCT) in patients undergoing hemodialysis (HD) remains unclear.

**Methods:**

We searched multiple databases (PubMed, EMBASE, and Cochrane Library) for studies published through August 2021 that evaluated the diagnostic performance of PCT in patients undergoing HD and having suspected bacterial infections. The bivariate fixed effects model was used to calculate pooled sensitivity, specificity, diagnostic odds ratio (DOR), positive likelihood ratio (PLR), negative likelihood ratio (NLR), and summary receiver operating characteristic (SROC) curves.

**Results:**

We identified a total of 1799 studies, of which seven diagnostic studies comprised 1444 patients and 430 bacterial infection episodes. Bivariate pooled sensitivity and specificity for PCT were 0.90 (95% CI: 0.85–0.94) and 0.83 (95% CI: 0.56–0.95), respectively. Furthermore, pooled DOR, PLR, NLR, and area under the curve (AUC) were 47 (95% CI: 11–209), 5.4 (95% CI: 1.7–16.9), 0.12 (95% CI: 0.07–0.20), and 0.92 (95% CI: 0.90–0.94), respectively. We also compared the diagnostic accuracy of PCT and C-reactive protein (CRP), and our results showed that the diagnostic accuracy parameters for PCT were significantly higher than those for CRP.

**Conclusions:**

PCT is a useful marker for diagnosis of bacterial infections in patients undergoing HD at a cutoff value of 1.5 ng/ml.

## Introduction

Patients undergoing hemodialysis (HD) are at an increased risk of bacterial infections, and they are associated with high morbidity and mortality rates [1]. The early diagnosis of such infections and timely targeted antibiotic therapy are thus pivotal for limiting morbidity rates, reducing costs, and improving patient condition; however, early diagnoses of bacterial infections remain challenging as conventional laboratory markers to detect them, such as white blood cell count, C-reactive protein (CRP) levels, and erythrocyte sedimentation rate, are often influenced by uremia, extracorporeal treatment, or immunosuppressive drugs [2,3].

Procalcitonin (PCT), a 116-amino acid precursor protein of calcitonin, has been reported to be an accurate and specific marker for the early diagnosis of bacterial infections in patients undergoing HD [4–11]. However, renal elimination is supposedly one of the major pathways for PCT eradication, and PCT release seems to be mediated by uremia or extracorporeal treatment. Moreover, elevated levels of baseline PCT have been found in a large number of chronic HD patients without any signs of infections [12–17]. A study reported that up to 44% of HD patients without bacterial infection had increased PCT levels (0.6–1.5 ng/ml) [17]. Furthermore, a recent study suggested that PCT could not effectively identify patients undergoing HD and having bacterial infections, because when the PCT cutoff value was ≥1 ng/ml, both diagnostic sensitivity and specificity were poor (77% and 59%, respectively) [18]. So far, PCT has not been extensively studied in patients undergoing HD; studies on this topic are characterized by either a relatively small sample size or have reported inconsistent findings. Therefore, the relative advantage of determining PCT levels in such patients is still unclear.

In this study, we performed a systematic review and meta-analysis with the aim of investigating the diagnostic accuracy of PCT in HD patients with bacterial infection.

## Methods

### Data sources, search strategy, and selection criteria

The systematic review and meta-analysis were performed following Preferred Reporting Items for Systematic Reviews and Meta-Analyses (PRISMA) guidelines [19]. We searched PubMed, EMBASE, and Cochrane Library for relevant studies. A search strategy was developed that involved using a combination of keywords and Medical Subject Headings/Emtree terms, which were ‘renal dialysis OR renal replacement therapy OR kidney failure OR renal insufficiency AND (sepsis OR “bacterial infection”) AND (procalcitonin OR PCT).’ We did not apply any language restriction to the electronic searches. The last search was performed on 21 August 2021. Studies were included on the basis of the following criteria: (1) evaluation of PCT alone or in combination with other laboratory markers, such as CRP, to diagnose bacterial infections (including Gram-positive and Gram-negative) in HD patients (2) presence of sufficient data to reconstruct a 2 × 2 contingency table for meta-analyses. A study was excluded if (1) it was a repeat of published articles (i.e., if the content or results were the same), (2) it reported incomplete data, (3) it studied patients with non-HD renal insufficiency such as patients with peritoneal dialysis, and (4) it was a theoretical study, case report, conference report, expert comment, systematic review, or meta-analysis. The literature search and study selection were performed by two reviewers (MT and WZ). Disagreements between reviewers were resolved by a discussion until a consensus is reached.

### Data extraction and quality assessment

Two reviewers independently performed data extraction and quality assessment (MT and WZ). Discrepancies, if any, were resolved by consensus. The following items were abstracted: author name, publication year, sample size, study design (e.g., prospective, retrospective), study population, outcome disease definition, timing of PCT measurement, use of markers other than PCT, cutoff levels of tested markers, and outcome data (true positive/negative values, false positive/negative values, sensitivity, and specificity). Quality assessment was performed using the Quality Assessment of Diagnostic Accuracy Studies (QUADAS-2) tool; the analyses were performed using RevMan version 5.2 (Cochrane Collaboration, Oxford, UK). QUADAS-2 consists of four sections, namely patient selection, index test, reference standard, and flow and timing.

### Statistical analysis

The bivariate fixed effects model was used to calculate pooled sensitivity, specificity, diagnostic odds ratio (DOR), positive likelihood ratio (PLR), negative likelihood ratio (NLR), and summary receiver operating characteristic (SROC) curves, as well as 95% CI; the analyses were performed using the MIDAS module for STATA software version 16.0 (Stata Corporation, College Station, TX). Threshold effects were calculated by testing Spearman correlation using Meta-DiSc version 1.4 (Cochrane Colloquium, Barcelona, Spain), with *p*<.05 indicating significance. *I*^2^ tests and a bivariate boxplot were used to test the heterogeneity of clinical trial results; if the *I*^2^ value was ≥50% and the chi-square test *p* value was ≤.1, the degree of heterogeneity was deemed to be significant. To explore the source of the heterogeneity, we performed subgroup analysis by study design characteristics. High- and low-cutoff values were defined as PCT levels ranging from 1.5 to 15.5 ng/ml and from 0.685 to 0.85 ng/ml, respectively. The Deek’s funnel plot was used to detect publication bias, with *p* < .05 indicating a strong bias. The visual presentation of diagnostic performance was assessed by the Fagan plot. We calculated kappa statistic to assess interobserver reliability between the two independent reviewers.

## Results

### Identification of studies

**Figure 1.**
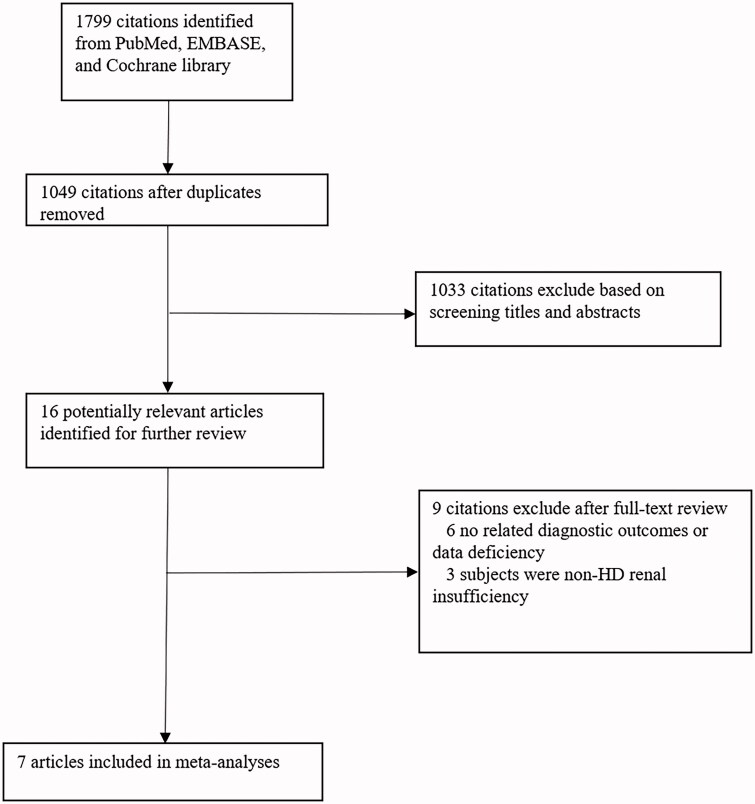
Flow chart depicting study identification and inclusion.

In total, 1799 studies were searched by the indices;1033 were excluded by screening the title and abstract, and 16 were shortlisted for further evaluation. Of these, six were excluded because of data deficiency or absence of related diagnostic outcomes [5,15,20–23] and three were excluded as they targeted PCT for diagnosing bacterial infections in patients with non-HD renal insufficiency [24–26]. Eventually, seven studies were included in the analysis (Figure 1). In total, the analysis comprised 1444 patients tested with PCT and 1279 patients tested with CRP, of which 430 (29.8%) and 392 (30.6%) patients were experiencing bacterial infections, respectively. The consistent results of the literature screening were: Kappa = 0.611, SE = 0.224, and *p*=.002.

### Study characteristics

One study included children with chronic HD [10], while other studies comprised adults. The means of diagnosis of infection could be classified into microbiologically documented infections (MDIs) and clinically documented infections (CDIs). MDIs were defined as positive results on microbiological culture of the blood, urine, respiratory tract samplings, and puncture or operative samplings. CDIs were defined as compatible clinical manifestations consistent with radiologic findings (computed tomography or ultrasonography), but negative culture results. The results showed that three studies confirmed the diagnosis of bacterial infections through diagnostic MDIs [6–8] and four studies confirmed the diagnosis of bacterial infections through MDIs and/or CDIs [9–11,18]. Table 1 lists the sensitivity and specificity of PCT/CRP tests. The sensitivity and specificity of PCT in identifying bacterial infections ranged from 76% to 100% and from 14.3% to 100%, respectively. Four studies also included CRP testing, with sensitivity ranging from 61% to 95% and specificity ranging from 47% to 90%. Four studies were prospectively conducted and three were retrospectively conducted. All studies described diagnostic cutoff thresholds for PCT or CRP. The cutoff thresholds widely varied (PCT, between >0.5 ng/ml and >15.5 ng/ml; CRP, between >5.0 mg/dl and >4.5 mg/dl). Table 1 lists the characteristics of all the seven included studies.

**Table 1.** Summary of the characteristics of the included studies.

Author, year, country	Study type	Gender Age(years) (M/F) mean(SD) /median(range)		Type of bacterial infection	Means of diagnosis of infection	Prevalence N (%)	Biomarkers tested	PCT, cutoff	PCT (%) sensitivity, specificity	CRP, cutoff	CRP (%) sensitivity, specificity
Mori, 2012, Germany	Prospective	42/34	69 (28–86)	Systemic or localized (pneumonia or arteriovenous graft infection)	MDI and CDI	15 (17)	PCT	0.5 ng/ml	86.7 96.7	NA	NA
Herget-Rosenthal, 2001, Germany	Prospective	NA	42 (35–57)	Sepsis, pneumonia, endocarditis pyelonephritis, invasive enterocolitis, other infections	MDI	36 (53)	PCT, CRP	1.5 ng/ml	89 81	5.0 mg/dl	89 48
Contou, 2014, France	Prospective	30/21	62 (51–72)	Bacteremia, urinary tract infection, respiratory tract infection, abdominal infection (including biliary tract infection, diverticulitis, peritonitis)	MDI and CDI	18 (35)	PCT, CRP	0.85 ng/ml	100 67	4.5 mg/dl	61 78
Fadel, 2016, Egypt	Retrospective	7/9	10.4 ± 4.2	Vascular access infections, pyelonephritis, pneumonia, enterocolitis, sepsis	MDI and CDI	16 (29)	PCT	0.5 ng/ml	80 14.3	NA	NA
Hamada, 2017, Egypt	Prospective	15/16	44.7 ± 2.1	Catheter-related bloodstream infection	MDI	16 (52)	PCT	15.5 ng/ml	94 100	NA	NA
Demir, 2018, Turkey	Retrospective	593/517	57.4 ± 14.6.	Sepsis, urinary tract infection, peritonitis, surgical wound infection, pneumonia	MDI	308 (28)	PCT, CRP	0.685 ng/ml	93 80	19.15 mg/dl	95 90
Schneider, 2019, Israel	Retrospective	29/24	66 ± 17	Endovascular infection, Clostridium difficile infection, skin and soft tissue infection, pneumonia, intra-abdominal infection	MDI and CDI	22 (40)	PCT, CRP	1.5 ng/ml	76 90	5.0 mg/dl	71 47

PCT: procalcitonin; CRP: C-reactive protein; MDI: microbiologically documented infection; CDI: clinically documented infection; NA: not available; M: males; F: females.

### Risk-of-bias assessments

**Figure 2.**
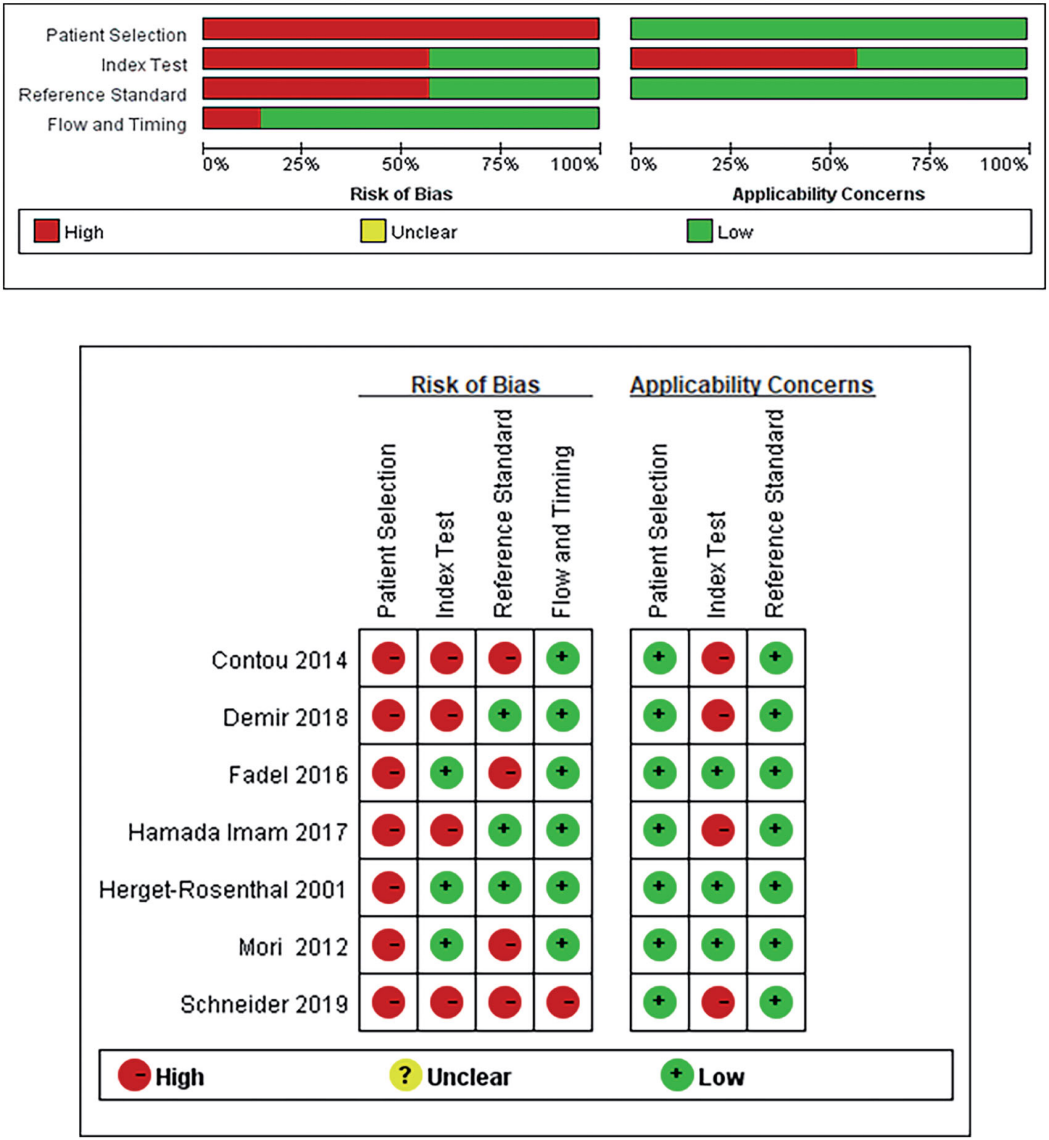
Risk of bias and applicability concerns.

Figure 2 shows the results of assessing the risk of bias by QUADAS-2. High risk of bias was indicated in the section of ‘patient selection,’ as the majority of the studies were case–control studies. Four studies were classified as having high bias in terms of ‘index test,’ owing to the lack of a clearly pre-specified cutoff threshold of PCT and CRP for a positive diagnosis. Four studies were classified as having high bias in terms of ‘reference standard,’ and one was classified as having high bias in terms of ‘flow and timing.’ There were no applicability concerns for all studies. The Deek’s funnel plot of the included studies suggested that there was no significant publication bias for PCT diagnostic outcomes (Supplementary Figure 1, *p*=.77).

### Threshold effect and heterogeneity

The Spearman correlation coefficient and *p* value were 0.143 and 0.760 for PCT and −0.400 and 0.600 for CRP, which indicated that there was no significant threshold effect; thus, we pooled sensitivity, specificity, PLR, NLR, DOR, and AUC. We used *I*^2^ and a bivariate boxplot (Supplementary Figure 2) to measure the heterogeneity. A substantial degree of heterogeneity was observed for PCT testing (*p*=.000, *I*^2^=88%) and for CRP testing (*p*=.004, *I*^2^=79%).

### Results of individual studies

**Figure 3.**
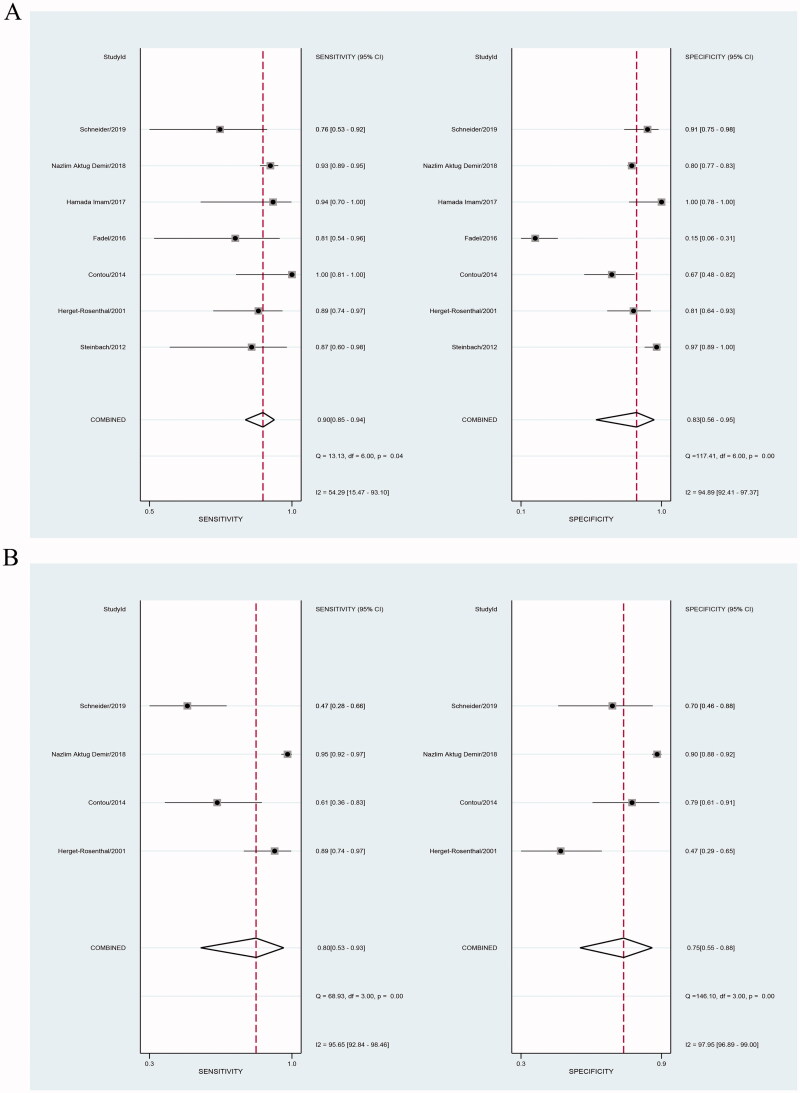
Forest plot of sensitivity and specificity for studies involving procalcitonin (PCT) (A) or C-reactive protein (CRP) (B) to detect bacterial infections in patients undergoing hemodialysis (HD).

**Figure 4.**
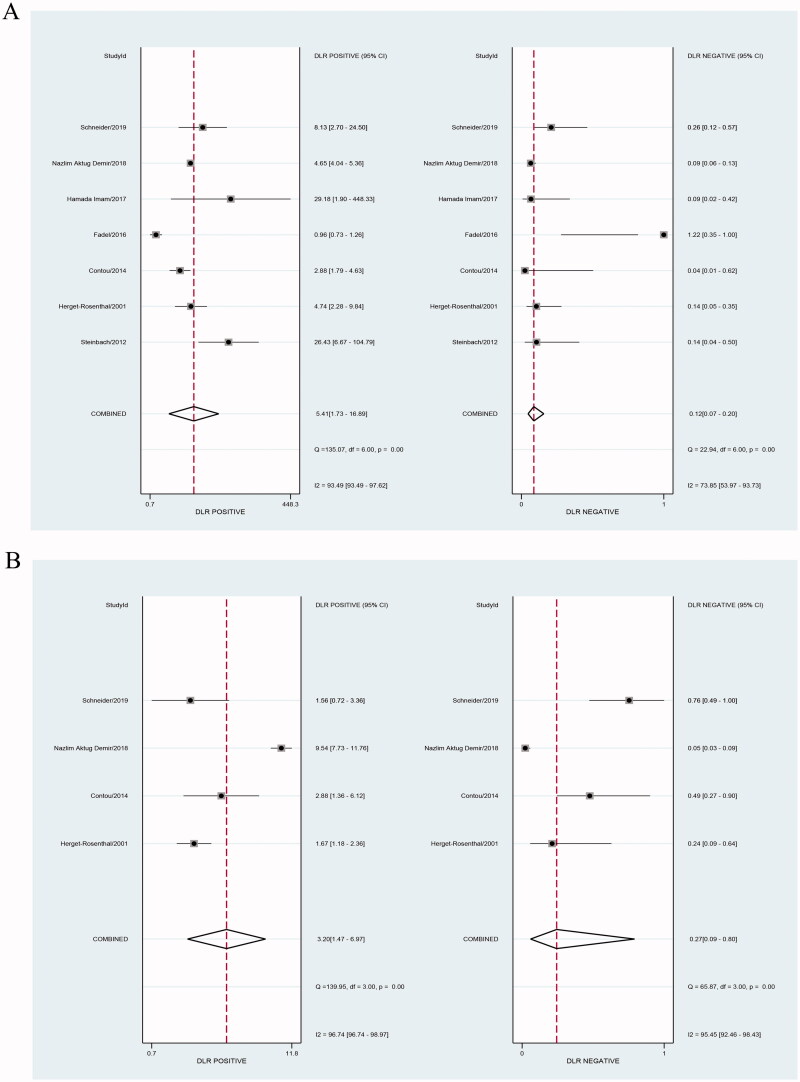
Forest plot of positive likelihood ratio (PLR) and negative likelihood ratio (NLR) for studies involving procalcitonin (PCT) (A) or C-reactive protein (CRP) (B).

**Figure 5.**
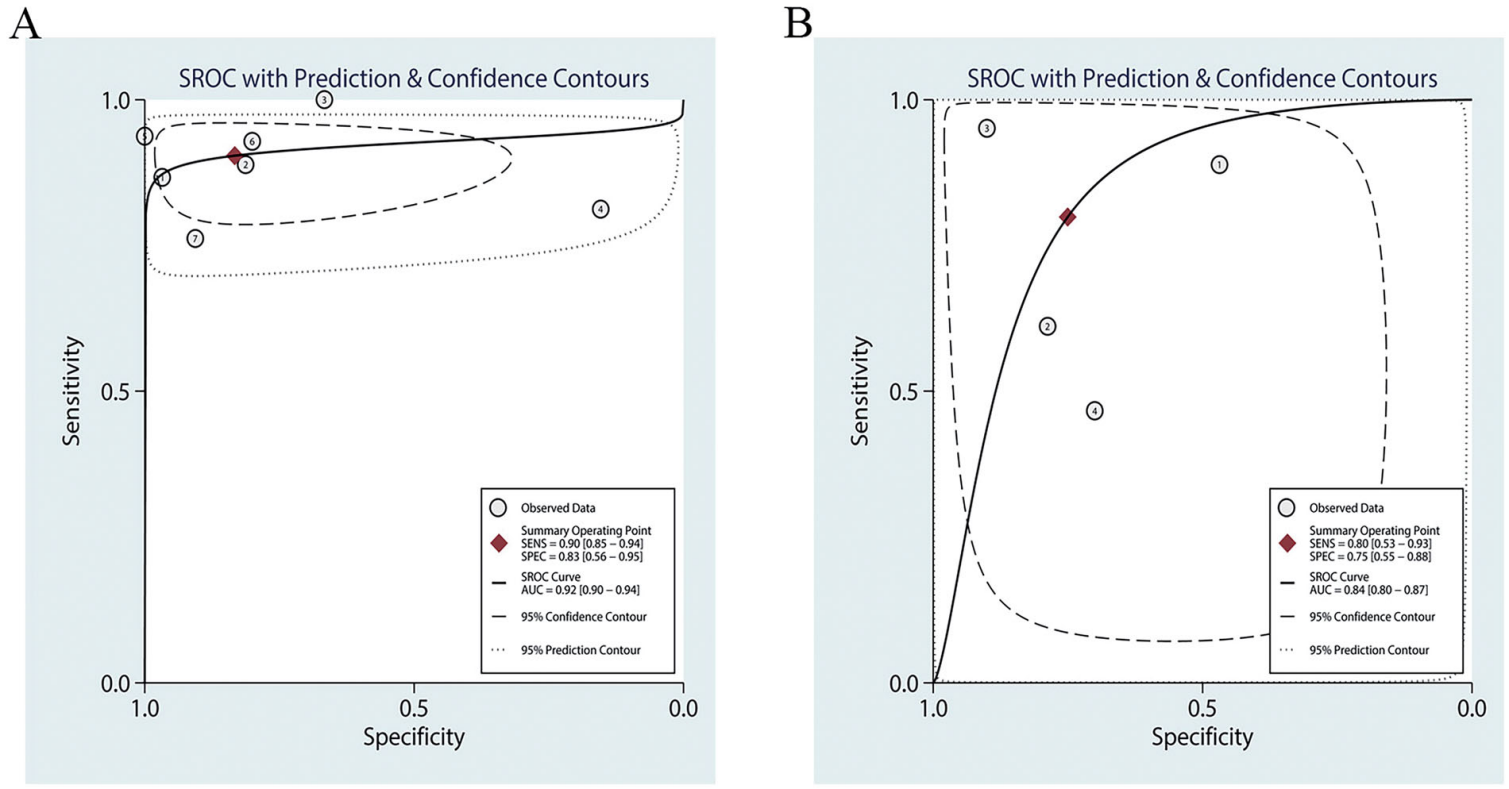
Summary receiver operating characteristic (SROC) curves for the diagnosis of bacterial infections in patients undergoing hemodialysis (HD). (A) Procalcitonin (PCT) and (B) C-reactive protein (CRP). AUC = area under the curve.

**Figure 6.**
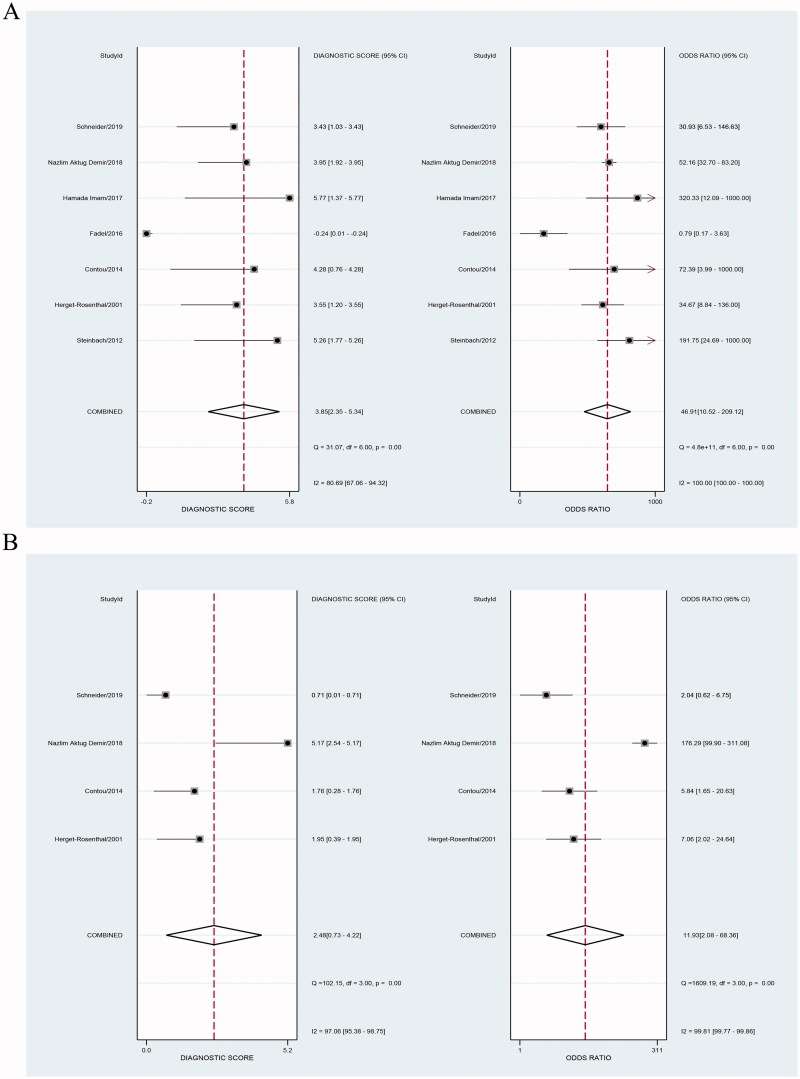
Forest plot of diagnostic odds ratio (OR) for studies involving procalcitonin (PCT) (A) or C-reactive protein (CRP) (B) to detect bacterial infections in patients undergoing hemodialysis (HD).

**Figure 7.**
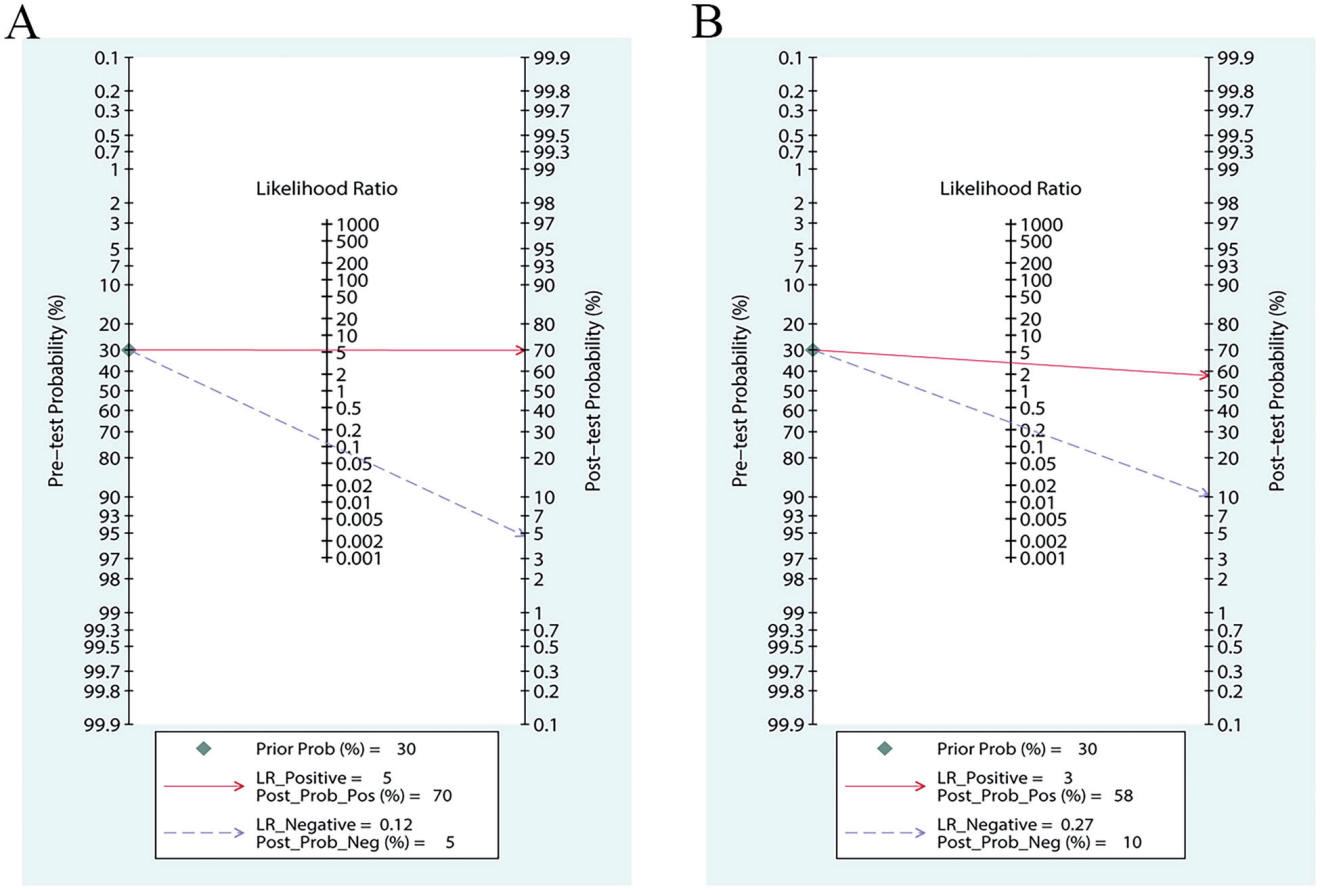
Fagan diagrams for (A) procalcitonin (PCT) and (B) C-reactive protein (CRP).

Our results showed that both PCT and CRP are more sensitive than specific in the diagnosis of bacterial infections in patients undergoing HD. Bivariate pooled sensitivity and specificity estimates for PCT were 0.90 (95% CI: 0.85–0.94) and 0.83 (95% CI: 0.56–0.95), respectively (Figure 3(A), Table 2), and those for CRP were 0.80 (95% CI: 0.53–0.93) and 0.75 (95% CI: 0.55–0.88), respectively (Figure 3(B), Table 2). The PLR for PCT (Figure 4(A), Table 2) was high enough to be used as a rule-in test (LR+: 5.4; 95% CI: 1.7–16.9), whereas the NLR was sufficiently low to be used as a rule-out test (LR−: 0.12; 95% CI: 0.07–0.20). On the other hand, the PLR for CRP (Figure 4(B), Table 2) was high (LR+: 3.2; 95% CI: 1.5–7.0), but the NLR was not low enough (LR−: 0.27; 95% CI: 0.09–0.80). To compare the overall performance of these two biomarkers independent of the threshold effect, we calculated the global measures of test performance: AUC and DOR. SROC showed an AUC of 0.92 (95% CI: 0.90–0.94) for PCT and 0.84 (95% CI: 0.80–0.87) for CRP (Figure 5). The DOR was 47 (95% CI: 11–209) for PCT and *1*^2^ (95% CI: 2–68) for CRP (Figure 6). Figure 7 shows the Fagan diagrams for both PCT and CRP. Based on the same pretest probability of 30%, the post-test probability for PCT (70%) was higher than that for CRP (58%), indicating that PCT testing is superior to CRP testing in this particular patient population.

**Table 2.** Summary of subgroup analysis of the included studies by study characteristics.

Variables	Number of studies	Sensitivity (95% CI)	Specificity (95% CI)	Likelihood ratio+ (95% CI)	Likelihood ratio- (95% CI)	AUC (95% CI)	Diagnostic OR (95% CI)
PCT							
Overall analysis	7	0.90 (0.85–0.94)	0.83 (0.56–0.95)	5.4 (1.7–16.9)	0.12 (0.07–0.20)	0.92 (0.90–0.94)	47 (11–209)
High cutoff value	3	0.86 (0.79–0.91)	0.89 (0.81–0.94)	7.8 (4.5–13.7)	0.16 (0.10–0.24)	0.92 (0.89–0.94)	51 (24–108)
Low cutoff value	2	0.95 (0.84–0.99)	0.77 (0.68–0.83)	4.1 (3.0–5.4)	0.06 (0.02–0.21)	0.91 (0.88–0.93)	68 (22–213)
Cutoff = 0.5 ng/ml	2	0.84 (0.72–0.92)	0.70 (0.14–0.97)	2.8 (0.4–18.5)	0.23 (0.07–0.69)	0.85 (0.81–0.88)	12 (1–228)
Outcome							
MDI	3	0.93 (0.90–0.95)	0.82 (0.54–0.94)	5.0 (1.7–15.2)	0.09 (0.05–0.16)	0.94 (0.92–0.96)	57 (11–296)
MDI and/or CDI	4	0.86 (0.73–0.94)	0.76 (0.31–0.96)	3.7 (0.8–16.4)	0.18 (0.08–0.41)	0.88 (0.85–0.91)	21 (3–167)
CRP							
Overall analysis	4	0.80 (0.53–0.93)	0.75 (0.55–0.88)	3.2 (1.5–7.0)	0.27 (0.09–0.80)	0.84 (0.80–0.87)	12 (2–68)

High cutoff value = 1.5–15.5 ng/ml; Low cutoff value = 0.685–0.85 ng/ml.

MDI: microbiologically documented infection; CDI: clinically documented infection; PCT: procalcitonin; CRP: C-reactive protein; OR: odds ratio; AUC: area under the curve.

### Subgroup analysis

We performed subgroup analysis by restricting studies with different cutoff values and outcome definitions (Table 2). For two studies [9,10] reporting test results using a standard PCT cutoff value (0.5 ng/ml), sensitivity, specificity, PLR, AUC, and DOR all markedly decreased as compared with the overall estimates. Moreover, two studies [6] reported diagnostic accuracy parameters using low cutoff values (0.685–0.85 ng/ml), sensitivity considerably increased (0.95, 95% CI: 0.84–0.99) and specificity correspondingly decreased (0.77, 95% CI: 0.68–0.83). In addition, three studies [7,8,18] reported diagnostic accuracy parameters using high PCT cutoff values (1.5–15.5 ng/ml), sensitivity markedly decreased (0.86, 95% CI: 0.79–0.91), and specificity correspondingly increased (0.89, 95% CI: 0.81–0.94). PLR and DOR considerably increased too.

Subgroup analysis using parameters based on the means of diagnosis of infection revealed that in comparison with the three studies [6–8] through MDIs confirmed bacterial infection dialysis patients, the four studies [9,10,18] confirmed by CDIs and MDIs showed appreciably decreased performance in sensitivity, specificity, NLR, AUC, and DOR. The diagnostic test accuracy indices for overall and subgroup analyses are summarized in Table 2.

### Sensitivity analysis

Sensitivity analysis was performed by eliminating one study at a time, and the effect of this elimination on the meta-analysis was evaluated. We observed that regardless of the excluded study, the combined DOR post-elimination did not significantly change, suggesting that the results of this analysis were not excessively dependent on one particular study. We thus believe that our findings are robust (Supplementary Figure 3).

## Discussion

The results of this meta-analysis confirmed that PCT had a high diagnostic accuracy, with AUC of 0.92, pooled sensitivity of 90%, and pooled specificity of 83% in patients undergoing HD and suspected of having an infection. Two previous meta-analyses studying the ability of PCT to diagnose bacterial infections in patients with normal renal function reported AUC ranging from 0.79 to 0.82, sensitivity of 76–88%, and specificity of 69–81% [27,28]. Herein we report that the diagnostic accuracy of PCT testing is better in patients undergoing HD than in individuals with normal renal function, which may be due to different cutoff values. Therefore, our meta-analysis initially established suitable cutoff values.

Serum PCT levels below 0.1 ng/ml are commonly detected in healthy individuals [29,30]. Cutoff values of >0.25 ng/ml have been used for distinguishing between bacterial infection and non-infection in patients with normal renal function [31,32]. However, recently, it has been reported that the median PCT in serum of HD patients without infectious diseases is 0.24 − 0.26 ng/ml [33,34], which is significantly higher than that of the general population. Therefore, the PCT cutoff level of 0.25 ng/ml in the general population would not be suitable for HD patients. In case of those receiving HD, PCT cutoff values are yet to be appropriately determined. Previous studies involving chronic HD patients indicated the appropriate cutoff of serum PCT levels to be 1.5 ng/ml [7,17]. However, several recent studies determined the PCT cutoff levels to be ≥0.5 ng/ml, which can be used to rule-in infection, while levels of <0.5 ng/ml can be used to rule-out infection in patients undergoing HD [9,10,32]. Here in our subgroup analysis revealed that in comparison with the cutoff value of 0.5 ng/ml, that of ≥1.5 ng/ml resulted in higher diagnostic accuracy parameters. Moreover, PCT levels ranging from 1.5 to 15.5 ng/ml had high specificity [0.89 (0.81–0.94)], whereas those ranging from 0.685 to 0.85 ng/ml had high sensitivity [0.95 (0.84–0.99)]. However, any threshold that increases sensitivity inversely decreases specificity, consequently resulting in unnecessary antibiotic usage. Due to the specificity significantly decreased (0.77, 95% CI: 0.68–0.83) using low cutoff values, for PCT, ≥1.5 ng/ml seems to be the most appropriate cutoff interval.

In addition, the timing of blood samples taken from HD to detect PCT is very essential. Recently, Kubo et al. [35] monitored the changes of PCT levels pre- and post-HD in 123 HD patients without bacterial infection. They found that after a single HD session, the PCT level decreased significantly from 0.23 to 0.12 ng/ml, indicating a PCT-removal rate by HD of 46.6%. This result suggests that pre-HD examination of PCT is crucial for accurately determining the bacterial infectious status in HD patients.

To the best of our knowledge, little data exist on evaluating the diagnostic value of PCT. One meta-analysis investigated the diagnostic value of PCT in patients with chronic renal insufficiency [36]; however, this study included patients with varying degrees of renal insufficiency and different dialysis methods, including peritoneal dialysis. Our study is more homogeneous and only targets chronic HD patients. Moreover, we performed a subgroup analysis based on the means of diagnosis of infection. The bacterial infections were determined by positive microbial culture and/or clinical inflammatory presentation. In comparison with the four studies including patients with a positive diagnosis but culture-negative (MDIs and/or CDIs), PCT has a higher diagnostic value in infected patients only with clear microbial record (MDIs). This can be reasonably explained because positive culture is the gold standard for diagnosing bacterial infections and the clinical signs for infection in HD patients are often subtle and nonspecific due to their compromised immune statuses, which caused CDIs have a poor diagnostic accuracy parameters.

CRP is an acute-phase reactant protein produced by the liver in response to inflammation, which is synthesized within 4–6 h after tissue injury or inflammation. CRP is in widespread clinical use as a general marker of inflammation but not specific for bacterial infections. Previous studies have revealed that it is of limited use for the discrimination of bacterial infection in patients with normal renal function; its diagnostic accuracy to identify bacterial infections is significantly lower than that of PCT [27,37]. In patients with different stages of renal disease, Sitter et al. [4] and Steinbach et al. [5] also observed that CRP levels are elevated and that CRP had low specificity (49%) for the diagnosis of bacterial infections. Our meta-analysis results are consistent with these observations – the diagnostic accuracy of PCT was indeed noted to be significantly higher than that of CRP.

This study had some limitations. First, we detected substantial heterogeneity between studies, but none of the study characteristics were responsible for the majority of this heterogeneity. The studies differed in several ways (e.g., methodological quality and clinical spectrum of patients). Thus, unrecorded differences between them could perhaps be held accountable for the heterogeneity. Second, we included only a few studies, particularly those on CRP, which prevented us from performing sensitivity analysis and publication bias. Last, this study was not registered online.

## Conclusions

The results of this meta-analysis confirmed a comparable diagnostic accuracy for PCT testing in patients undergoing HD and having a bacterial infection using the cutoff value of ≥1.5 ng/ml; Moreover, the diagnostic accuracy of PCT was significantly higher than that of CRP. However, our study results should be carefully and cautiously interpreted, as only a limited number of studies were included and a high level of heterogeneity was found between them. Further research is thus warranted.

## Data Availability

The data of this study are available from the corresponding author. Professor Qiang He conceived and designed the meta-analysis; Mei Tao and Wei Zhang searched literatures and extracted data; Danna Zheng and Xudong Liang analyzed the data. All authors reviewed the manuscript and approved the submitted version.
